# In Silico Study of Different Thrombolytic Agents for Fibrinolysis in Acute Ischemic Stroke

**DOI:** 10.3390/pharmaceutics15030797

**Published:** 2023-02-28

**Authors:** Yilin Yang, Boram Gu, Xiao Yun Xu

**Affiliations:** 1 Department of Chemical Engineering, Imperial College London, South Kensington Campus, London SW7 2AZ, UK; 2 School of Chemical Engineering, Chonnam National University, 77 Yongbong-ro, Buk-gu, Gwangju 61186, Republic of Korea

**Keywords:** thrombolysis, acute ischemic stroke, tissue plasminogen activator, tenecteplase, reteplase, alteplase, urokinase, pharmacokinetics, pharmacodynamics

## Abstract

Alteplase is the only FDA-approved drug for thrombolysis in acute ischemic stroke (AIS). Meanwhile, several thrombolytic drugs are deemed to be promising candidates to substitute alteplase. This paper evaluates the efficacy and safety of urokinase, ateplase, tenecteplase, and reteplase for intravenous AIS therapy by computational simulations of the pharmacokinetics and pharmacodynamics combined with a local fibrinolysis model. The performances of the drugs are evaluated by comparing clot lysis time, plasminogen activator inhibitor (PAI) inhibition resistance, intracranial hemorrhage (ICH) risk, and activation time from drug administration to clot lysis. Our results reveal that urokinase has the quickest lysis completion but the highest ICH risk due to excess fibrinogen depletion in systemic plasma. While tenecteplase and alteplase have very similar thrombolysis efficacy, tenecteplase has a lower risk of ICH and better resistance to PAI-1. Among the four simulated drugs, reteplase has the slowest fibrinolysis rate, but fibrinogen concentration in systemic plasma is unaffected during thrombolysis.

## 1. Introduction

**Figure 1:**
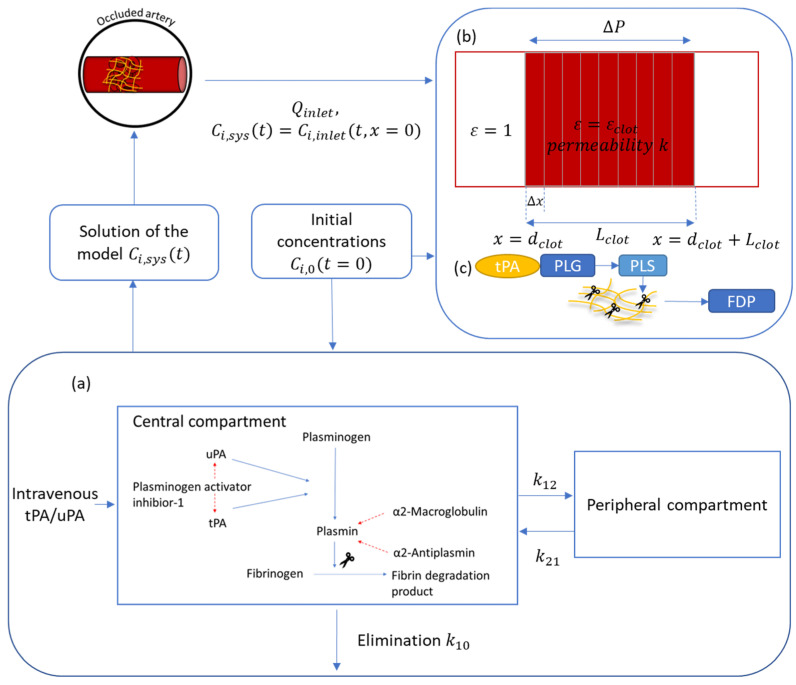
Overview of the 1D thrombolytic model. (**a**) Two-compartment PK-PD model. (**b**) Schematic of local PD model which couples 1D blood flow (in *x*-direction) with species transport and fibrinolytic reactions. The model solves clot lysis in an occluded artery where the red area indicates the clot with a porosity of *ε_clot_* and the open area is clot-free with ε=1. (**c**) Fibrinolysis mechanism on fibrin.

Thrombus is the final product of the hemostasis process to repair damaged blood vessels. It is made of platelets, cellular components, and fibrin fiber [1]. Unregulated thrombus formation can result in the blockage of blood circulation and thus cause serious diseases, such as ischemic stroke, myocardial infarction, and pulmonary embolism [2]. One of the well-established treatments for these diseases is thrombolytic therapy. Thrombolytics are a group of medications which can lyse the intravascular thrombus and restore blood flow by catalyzation of the serine protease plasminogen to plasmin that dissolves the crosslinking fibrin in the clot. The detailed thrombolysis mechanism is shown in Figure 1a. The first generation of thrombolytic drugs, streptokinase and urokinase, are fibrin-unspecific agents [3]. They quickly bind with plasminogen in the plasma and the clot. This may result in excess depletion of plasminogen and a high risk of intracranial hemorrhage (ICH) during stroke treatment [2]. The second-generation agents, such as tissue plasminogen activator (tPA) and its variants, are fibrin-specific [3]. These serine proteases form a ternary complex with plasminogen on the fibrin surface before cleaving the plasminogen to plasmin. They have a low affinity to the substrate of plasminogen in the absence of fibrin. Theoretically, localized fibrinolysis can reduce the systematic effect and the associated internal hemorrhage risk [2].

Compared with other arterial ischemic diseases, the application of thrombolytic drugs in ischemic stroke treatment faces more challenges. Early clinical studies of traditional streptokinase and urokinase intravenous (IV) therapies for acute ischemic stroke (AIS) patients show high mortality rates and insignificant improvement in recanalization rates when compared with control groups [4,5,6,7]. At present, alteplase is the only approved thrombolytic drug for standard-of-care AIS treatment authorized by the U.S. Food and Drug Administration (FDA) [8]. It is a recombinant tPA that has high fibrin specificity in theory. The milestone clinical trial from the National Institute of Neurological Disorders and Stroke (NINDS) group was the first to show significantly improved functional outcomes after 90 days of intravenous tPA therapy in stroke patients. However, subsequent clinical studies and meta-analysis indicate that the benefit of alteplase is undermined by plasminogen activator inhibitor-1 (PAI-1), a short half-life, and increasing probability of ICH.

Genetically engineered tPA variants, such as reteplase and tenecteplase, are promising drugs aiming to increase fibrin affinity and extend half-life [9,10,11,12,13]. Nevertheless, none of these tPA variants has shown better functional clinical outcomes than alteplase for AIS treatment so far [11,14]. Recent clinical research from developing countries found that IV urokinase and its precursor, pro-urokinase, seem to have similar efficacy and safety to alteplase in treating mild to moderate AIS, while the cost of urokinase is only one-tenth of that of alteplase [15,16,17,18,19,20]. These contested clinical results did not fully reflect the theoretical advantage of drugs as scientists expected. Therefore, there is a need to systematically compare the efficacy and safety of drugs based on their unique mechanism of action and drug transport processes. This requires a deep understanding of the pharmacokinetics and pharmacodynamics (PK-PD) of these drugs.

Thrombolysis is a complex process. The efficacy of drugs depends on the mechanism of action, reaction kinetics, blood flow, body exposure to the drugs, and individual differences. A mathematical model combining systemic PK-PD models (reaction kinetics in plasma) with the local clot lysis process can be useful in such a situation. To the best of our knowledge, there is no published study comparing the efficacy of thrombolytic drugs via in silico modeling. So far, the most detailed computational thrombolysis model is the 3D patient-specific model developed by Piebalgs et al. [21], which was further extended by Gu et al. who also developed a reduced-order model for better computational efficiency [22].

In the present study, the 1D two-compartment PK-PD model of Gu et al. [22] was adopted and modified to simulate intravenous thrombolysis of four known thrombolytic drugs, alteplase, urokinase, tenecteplase, and reteplase, in a simplified model of the middle cerebral artery with an occluding clot. Temporal evolutions of lysis proteases in plasma and the clot were analyzed. The performances of the drugs were evaluated by comparing clot lysis time, PAI-1 inhibition resistance, ICH risk, and activation time from administration to clot lysis. These parameters were chosen because clot lysis time is directly related to the efficacy of thrombolytic drugs, while PAI-1 inhibition resistance determines the real amount of drugs in the body that can be effective for clot lysis. ICH risk was assessed by monitoring plasma FBG concentration. Activation time indicates the time needed for each drug to start the clot lysis.

## 2. Materials and Methods

### 2.1. Drug Properties, Dose Regimen, and Reaction Kinetics

Compared with alteplase, tenecteplase has high fibrin affinity, and a longer plasma half-life. Tenecteplase also has a high resistance to PAI-1. The suggested dose regimen for AIS is a quick one-bolus IV infusion (0.25 mg/kg) within 5 s [11].

Reteplase demonstrates weaker binding with fibrin than native tPA does and consequently allows more free diffusion through the clot. It also has a low affinity to plasminogen but a longer half-life. Clinical research on IV reteplase therapy for AIS is very limited; hence, the recommended dose regimen for acute myocardial infarction was chosen for simulation and comparison. This includes a double bolus of 10U (17.4 mg) each over 2 min with an interval of 30 min [12].

Urokinase (uPA) directly cleaves plasminogen to plasmin. It has no affinity or specificity to fibrin. It can be rapidly inhibited by plasminogen activator inhibitors. The dose regimen reported in a recent clinical study is 1–1,500,000 U or 14,300–20,000 U/kg. Here, we chose 1,500,000 U (about 11.3 mg) IV infusion over 30 min [20,23].

Alteplase has high affinity to fibrin and plasminogen but a short half-life of only about 5 min. The standard dose is 0.9 mg/kg, with 10% of the total being administered as initial IV bolus in 1 min, and the remaining 90% infused over 60 min [24].

For different plasminogen activators, the reactions between plasminogen, plasmin, and activators in the plasma phase are described by the Michaelis–Menten kinetics:

(1) $$\mathrm{tPA}/\mathrm{uPA}+\mathrm{PLG}\overset{\;K_{M,PLG}\;\&\;k_{cat,PLG}\;}{\to }\mathrm{tPA}+\mathrm{PLS},r_{tPA-PLG}=\frac{k_{cat,PLG}C_{tPA/uPA\;}C_{PLG}}{K_{M,PLG}+C_{PLG}}$$

where $C_{tPA/uPA}$, $C_{PLG}$ is the concentration of plasminogen activators and plasminogen in the plasma phase, and *K_M_* and *k_cat_* are the Michaelis constant and catalytical constant. Values for theses parameters were obtained from in vitro experiments in the literature [12,23,24,25,26,27].

The inhibition effect of PAI-1 is described by:

(2) $$\mathrm{tPA}/\mathrm{uPA}+\mathrm{PAI}\overset{\;k_{PAI}\;}{\to }\mathrm{inactive},r_{PAI}=k_{PAI}C_{tPA}C_{PAI}$$

where $k_{PAI}$ is the second-order reaction constant for reaction between PAI-1 and tPA/uPA. The PK parameters $k_{cp},\;k_{pc},\;and\;k_{el}$ were obtained from clinical data in the published literature. Kinetic parameters involved in the above reactions are summarized in Table 1.

The clot-phase reaction kinetics are associated with the fibrin (F) affinity and tPA/uPA catalyzation of plasminogen. The reactions are described by the Michaelis–Menten equation.

For tPA:

(3) $$\mathrm{tPA}+F\overset{\;k_{a,tPA}k_{d,tPA}\;}{⇄}\mathrm{tPA}-F,\;r_{tPA-F}=k_{a,tPA}C_{tPA}n_{free}-k_{d,tPA}n_{tPA}$$

(4) $$\mathrm{tPA}-F+\mathrm{PLG}-F\overset{\;K_{M}\;\&\;k_{M,cat}\;}{\to }\mathrm{tPA}-F+\mathrm{PLS}-F,\;r_{PLS,MM}=\frac{k_{cat}n_{PLG}n_{tPA}}{K_{M}\left(1-\varepsilon_{clot}\right)+n_{PLG}}\;\;$$

Note that uPA has no affinity with fibrin. Therefore, we assumed that unbound uPA reacts directly with plasminogen on fibrin:

(5) $$\;\mathrm{uPA}+\mathrm{PLG}\cdot F\overset{K_{M},k_{cat}}{\to }\mathrm{uPA}+\mathrm{PLS}\cdot F,\;r_{pls,MM}=\frac{k_{cat}\;n_{PLG}C_{uPA}}{K_{M}\left(1-\varepsilon_{clot}\right)+n_{PLG}}$$

where $K_{M}$ and *k_cat_* are the Michaelis constant and catalytical constant. K_d_ is the dissociation constant between fibrin and plasminogen activators, which is the ratio of desorption constant $k_{d}$ to adsorption constant $k_{a}$. $n_{free}$ is the free binding site. $n_{i}$ is the concentration of bound species. $\varepsilon_{clot}$ is the porosity of the clot. Values for the kinetic parameters for the four simulated drugs are given in Table 2.

### 2.2. Mathematical Model

The mathematical modeling platform developed by Gu et al. [22] was adopted in this study. As shown in Figure 1, this model comprises two sub-models: a systemic PK-PD model and a local PD model. The PK-PD model describes reactions between lysis proteins in systemic plasma, while the local PD model describes the transport of lysis proteins and the associated fibrinolytic reactions in the artery where the clot is located. The temporal concentration profile of the active species calculated from the PK-PD model provides the inlet boundary condition for the local PD model. In the systemic PK-PD model, the mass balance equation for a thrombolytic drug is written as follows:

(6) $$\frac{dC_{c}}{dt}=\frac{I}{V_{c}M_{w,PA}\;}-k_{el,D}C_{c}-k_{cp}C_{c}+k_{pc}C_{p}+S_{PA}+R_{d}^{plasma}$$

where $V_{c}$ is the distribution volume of central compartment when the patient is assumed to have a body weight of 80 kg,$\;M_{w,PA}$ is the molecular weight of plasminogen activators,$\;k_{el,D}$ is the elimination constant, $C_{c}$ is the concentration in the central compartment, and $k_{cp}$ and $k_{pc}$ are the distribution constants for the central compartment and peripheral compartment, respectively. $S_{PA}$ is the secretion of plasminogen activators, which should be zero for tPA mutants:

(7) $$\;S_{\mathrm{Tenecteplase}/\mathrm{Reteplase}}=0$$

Temporal concentration change of other fibrinolytic proteins in the systematic model ($C_{i,sys})$ is calculated as follows:

(8) $$\frac{dC_{i,sys}}{dt}=-k_{el_{i}}C_{i,sys}+R_{i}^{plsma}+S_{i}\;,\;i=PLG,PLS,\;AP,\;FBG.\;MG\;and\;PAI$$

where $k_{el_{i}}$ is the elimination constant, and $S_{i}$ is the systematic secretion of other proteins, *i*. $R_{i}^{Plasma}$ represents the fibrinolysis reactions in plasma.

The temporal concentration change of drugs in the peripheral compartment ($C_{p})$ is calculated as:

(9) $$\frac{dC_{p}}{\mathrm{dt}}=k_{cp}C_{c}-k_{pc}C_{p}\;$$

The fibrinolysis reaction mechanism is shown in Figure 1. Six fibrinolysis proteins are involved in the reactions, including plasminogen (PLG), plasmin (PLS), plasminogen activator inhibitior-1 (PAI-1), α2-antiplasmin (AP), and α2-macroglobulin (MG).

As shown in Figure 1b, the local PD model treats the clot as a porous medium that has a length of *L_clot_* along the direction of flow. Transport of species in the clot is governed by the convection–diffusion–reaction equation in the *x*-direction:

(10) $$\frac{\partial \varepsilon n_{i}}{\partial t}=-\frac{\partial \varepsilon Un_{i}}{\partial x}+D_{i}\frac{\partial^{2}\varepsilon Un_{i}}{\partial x^{2}}+\varepsilon R_{i}^{total},\;\;\;\;R_{i}^{total}=R_{i}^{plasma}+R_{i}^{clot}$$

where $R_{i}^{clot}$ is the clot-phase reaction rate, $R_{i}^{plasma}$ is the plasma-phase reaction rate, and $R_{i}^{total}$ is the total reaction rate. $D_{i}$ is the drug diffusivity in the clot, and ε is the porosity of the clot. U is the flow velocity in the clot, which is calculated from Darcy’s law:

(11) $$U=\frac{k}{\mu }∆p$$

where *k* is the permeability of the clot in unit $m^{2}$. It is calculated from the Davis equation, and further details can be found in Piebalgs et al. [18]. μ is the dynamic visocity in unit Pa·S, and ·p is the pressure drop per unit length of the clot. The degradation of binding sites by PLS is calculated as:

(12) $$\frac{\partial n_{total}}{\partial t}=-k_{deg}\gamma n_{PLS}$$

where $k_{deg}$ is the lysis coefficient, and γ is the number of cuts to degrade one fibrin unit. Detailed information about the model and numerical procedure can be found in Gu et al. [22].

### 2.3. Additional Kinetic Parameters and Model Validation

Apart from the parameters mentioned above, kinetic parameters for reactions between the plasmin, antiplasmin, macroglobulin, and fibrinogen can be found in our previous study [22]. Results from the systemic PK model of urokinase, tenecteplase, and reteplase were compared with clinical data available in the literature [31,37,38], while validation for the PK model of alteplase can be found in our previous study [21,22]. We chose to compare the systemic concentrations of fibrinogen, plasminogen, and plasminogen activators for model validation due to the accessibility of data. Detailed comparisons can be found in Appendix A.

## 3. Results

### 3.1. Comparison of Therapeutic Efficacy

**Figure 2:**
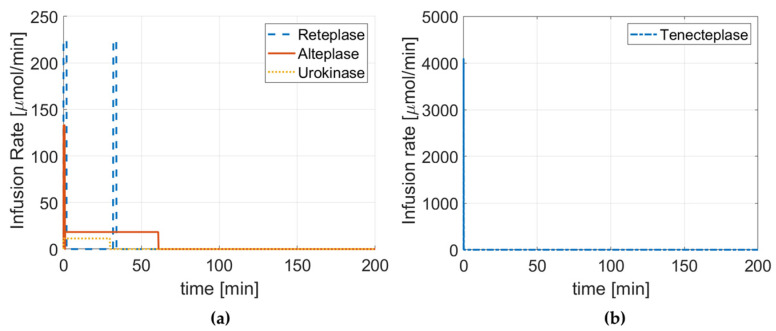
Intravenous dose regimen for (**a**) reteplase, alteplase, and urokinase, and (**b**) tenecteplase.

**Figure 3:**
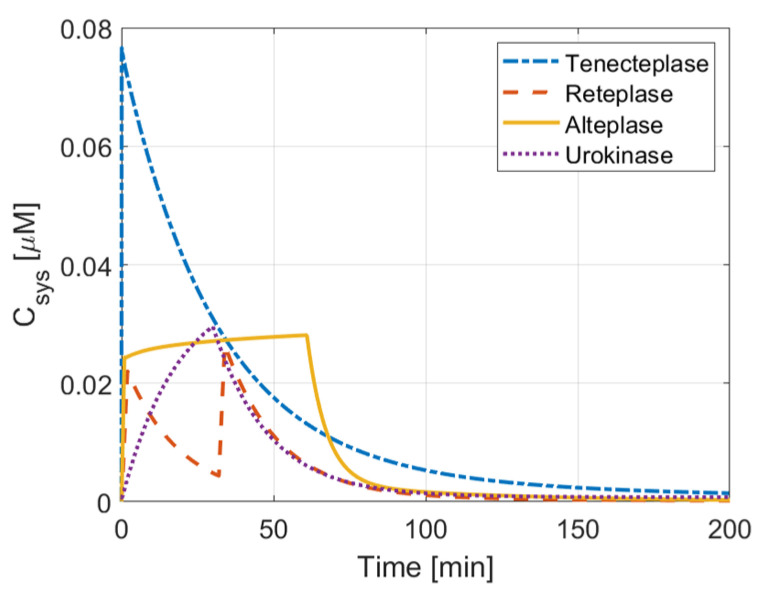
Systemic concentration profiles of different thrombolytic drugs.

**Figure 4:**
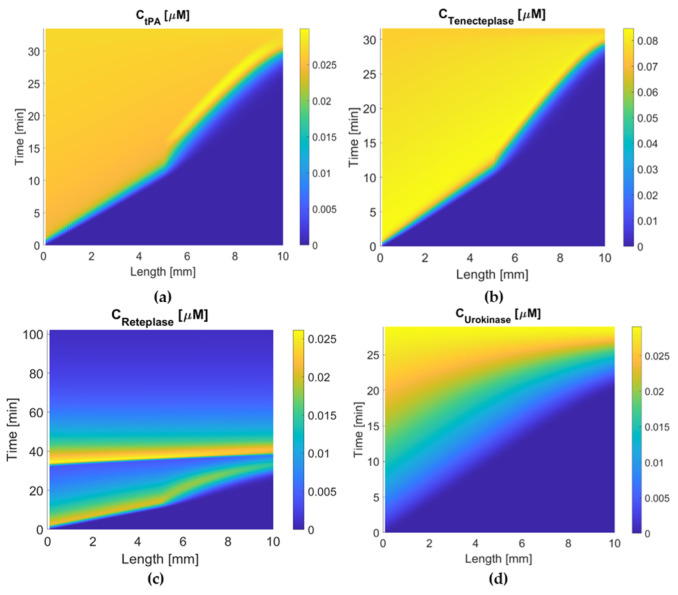
Temporal and spatial concentration profiles for (**a**) alteplase, (**b**) tenecteplase, (**c**) reteplase, and (**d**) urokinase in the clot. Lysis time: urokinase (26 min) < tenecteplase (30 min) ≈ alteplase (30 min) < reteplase (100 min).

Figure 2 summarizes the dose regimen of each drug, while Figure 3 and Figure 4 illustrate the concentration of four drugs in the central compartment and local PD model, respectively.

It can be seen that the large bolus amount (20 mg), combined with the slow clearance of tenecteplase, provides the greatest drug exposure (area under the curve in Figure 3) among the four drugs. Compared to alteplase, tenecteplase has a similar lysis time and concentration profile at the clot front, suggesting comparable therapeutic efficacy between the two drugs (Figure 4). At the same time, it is worth noting that the total dose of tenecteplase (0.25 mg/kg) is significantly lower than that of alteplase (0.9 mg/kg), which indicates the high effectiveness of tenecteplase over alteplase. Urokinase has the shortest lysis time, while the total drug exposure is less than that of alteplase. The dose amount of urokinase is also the lowest among the four simulated drugs. These properties indicate its high therapeutic efficacy for thrombolysis. In contrast, the large dose of reteplase is quickly eliminated from the body (Figure 3). Its peak concentration and total body exposure are the lowest among the four drugs, resulting in a low spatial and temporal drug concentration in the clot, especially after 60 min. The sluggish reaction kinetics further reduce the lysis rate.

### 3.2. ICH Risk

**Figure 5:**
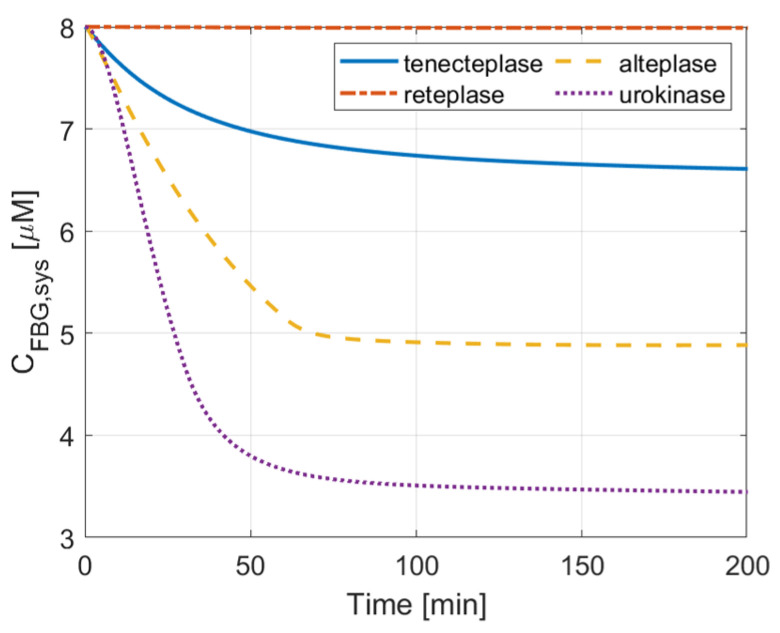
Comparison of fibrinogen concentration in systemic plasma. FBG concentration in the plasma (lower concentration represents higher ICH risk): urokinase < alteplase < tenecteplase < reteplase.

**Figure 6:**
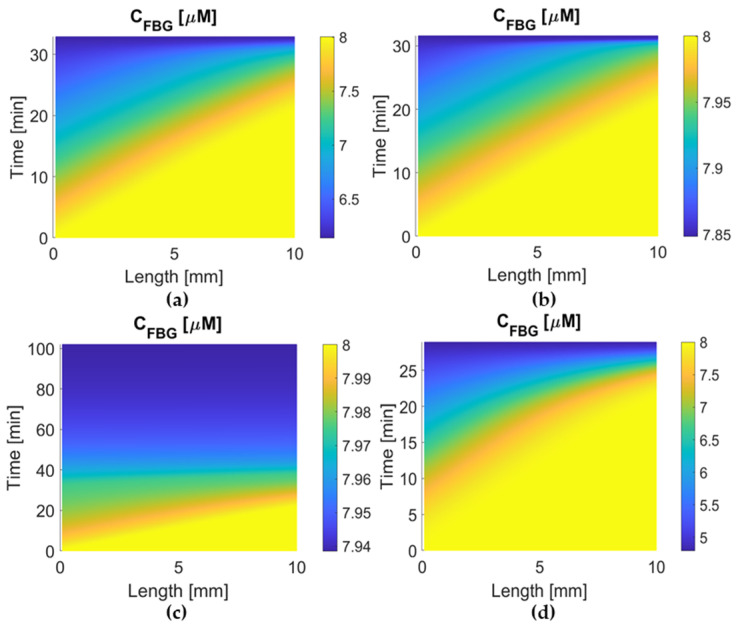
Temporal and spatial concentration profiles of free-phase fibrinogen for (**a**) alteplase, (**b**) tenecteplase, (**c**) reteplase, and (**d**) urokinase in the clot. Free-phase FBG concentration in the clot (lower concentration represents higher ICH risk): urokinase < alteplase < tenecteplase < reteplase.

Since low fibrinogen (FBG) concentrations can increase the ICH risk, comparisons of FBG concentration were made for the four simulated drugs, as shown in Figure 5 and Figure 6 for FBG concentrations in the systemic PK-PD model and local PD model, respectively. It can be clearly seen that urokinase causes a rapid depletion and the lowest level of FBG concentration in systemic plasma (Figure 5), but local FBG concentration within the clot only falls slightly during urokinase therapy (Figure 6d). Tenecteplase therapy can maintain higher levels of FBG concentration than alteplase, whereas the FBG concentration after reteplase therapy is almost unchanged in both the plasma and clot, which is due to its low affinity to fibrin and sluggish reaction kinetics.

### 3.3. Activation Time and Extent of Lysis

**Figure 7:**
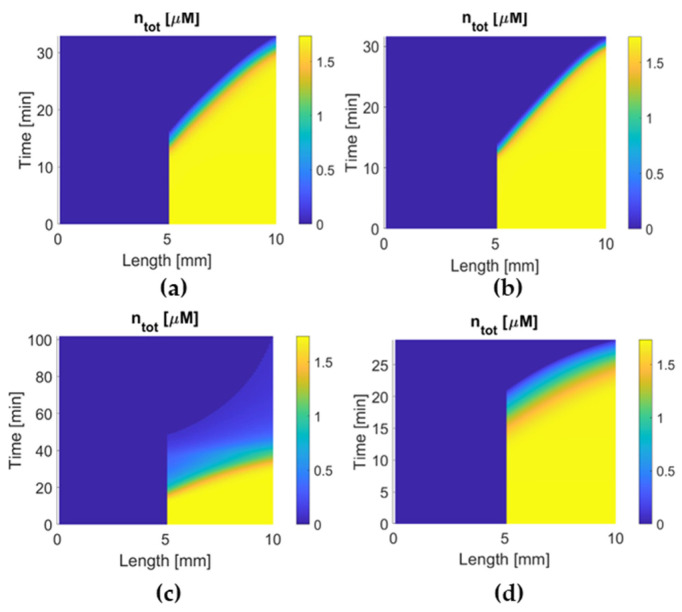
Temporal and spatial concentration profiles of binding sites for (**a**) alteplase, (**b**)tenecteplase, (**c**) reteplase, and (**d**) urokinase in the clot. Activation time to start lysis: tenecteplase (12 min) ≈ alteplase (12 min) < urokinase (16 min) < reteplase (18 min).

Concentration maps of the binding site in the clot are shown in Figure 7 where the time that the clot starts to dissolve (the time from yellow to blue at 5 mm along the length) can be found. Both alteplase and tenecteplase have short activation times, while reteplase and urokinase take much longer time to activate clot lysis. This is associated with the free-phase concentration, fibrin affinity, and reaction activity of the drug. After lysis is activated at the clot front, tenecteplase and alteplase dissolve binding sites rapidly within 2 min. In comparison, it takes 10 min for urokinase and over 60 min for reteplase, which can be explained by slow plasminogen activation and low local concentration at the clot.

### 3.4. PAI-1 Inhibition Effect

**Figure 8:**
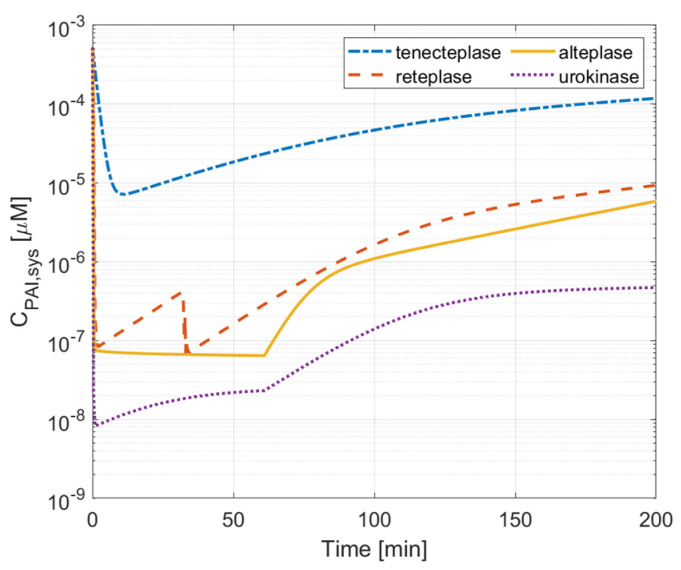
Comparison of PAI-1 systematic concentrations.

**Figure 9:**
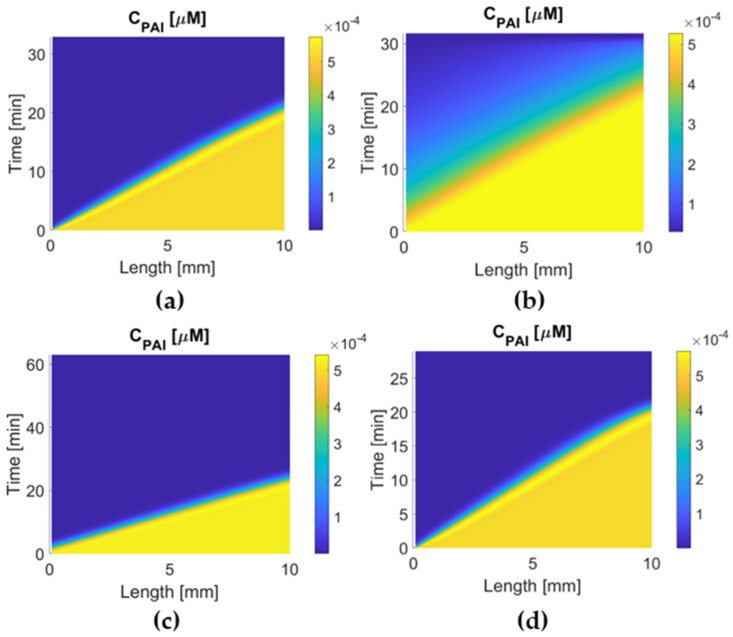
Temporal and spatial concentration profiles of PAI-1 for (**a**) alteplase, (**b**) tenecteplase, (**c**) reteplase, and (**d**) urokinase in the clot.

Figure 8 and Figure 9 compare the variation in PAI-1 concentration over time after drug administration. A higher concentration of PAI-1 indicates a better resistance to PAI-1 inhibition. The results show clearly that the highest systematic concentration of PAI-1 is maintained with tenecteplase therapy where the initial fall in PAI-1 is less dramatic compared to the other drugs, and it recovers to a high level, indicating that tenecteplase has high resistance to PAI-1. For alteplase and reteplase, the systematic PAI-1 concentration drops dramatically to about $10^{-7}\;\mu M$ after 1 min of drug administration. Urokinase causes the lowest plasma PAI-1 concentration. In Figure 9, the temporal and spatial PAI-1 concentration profiles in the clot are similar for alteplase, reteplase, and urokinase, where PAI-1 is quickly depleted to about $1\times 10^{-6}\;\mu M$ in a few minutes after reacting with the drug. For tenecteplase, the depletion of PAI-1 is much slower, and the concentration of PAI-1 is about $5\times 10^{5}\;\mu M$ when the clot is lysed completely. The performance of the four drugs in PAI-1 inhibition resistance is ranked as follows: urokinase < alteplase ≈ reteplase < tenecteplase.

## 4. Discussion

In this study, we simulated the thrombolysis process of four thrombolytic drugs in an idealized, fully occluded middle cerebral artery. The spatial and temporal variations of fibrinolytic proteins can be used to assess the efficacy and ICH risk of each therapy. Qualitative comparisons were made between our simulation results and relevant findings reported in the literature. In recent years, several studies reported comparable treatment outcomes between alteplase and urokinase [17,20,23]. In vitro experiments show that the clot lysis rate of urokinase is higher than that of alteplase [39]. Our simulation results show that urokinase has the shortest lysis time (about 26 min) among the four drugs, but the total systemic body exposure is less than that of alteplase. The high concentration, low clearance, and fast reaction kinetics of urokinase with PLG provide a good therapeutic efficacy and short time to start lysis. The well-known complication of high bleeding risk is also revealed in our simulation via low FBG concentration in the urokinase therapy. Its non-specific catalyzation of PLG decreases the systemic FBG concentration rapidly in the central compartment (Figure 5), which indicates a higher risk of ICH. Urokinase can also be quickly inhibited by PAI-1, which is demonstrated by the lowest PAI concentration in systemic plasma and within the clot among the four drugs (Figure 8 and Figure 9d).

Tenecteplase (TNK-tPA) is a mutation of alteplase with the substitution of T103N (introducing glycosylation site), N117Q (deleting glycosylation site), and Lys296-His297-Arg298-Arg299 with four alanines [10]. It has a longer plasma half-life and 15-fold higher fibrin specificity than alteplase [11]. It also has less impairment of hemostasis and greater resistance to PAI-1. Tenecteplase has been recommended as an alternative to alteplase in the US due to its low ICH risk and simple single-bolus administration [40]. Some studies have shown that tenecteplase may outperform alteplase or urokinase in terms of treatment outcome or recanalization rate [41,42]. Using our simulation model, the highest initial dose of tenecteplase, combined with the slowest drug clearance among the four drugs, gives rise to the highest plasma concentration in the body. Compared to alteplase, tenecteplase has a very similar clot lysis and activation time, but a much lower ICH risk (higher systematic concentration and free-phase concentration of FBG in Figure 5 and Figure 6b). Moreover, Figure 8 reveals that tenecteplase also has an excellent resistance to PAI-1 inhibition. These properties indicate that tenecteplase might be a promising candidate for AIS treatment.

Reteplase is a modified version of tPA with a longer half-life due to the absence of epidermal growth factor and fibronectin finger domains. However, it has a low affinity to fibrin because of the deletion of the fibronectin finger region. An in vitro experiment shows that reteplase has a faster clot lysis rate than alteplase [43]. However, clinical studies of reteplase treatment for AIS did not show any additional benefit in any aspect when compared with alteplase [12,44]. Our simulation results show that reteplase has the longest lysis time (100 min) and the lowest temporal concentration due to the sluggish reaction kinetics and shorter half-life than urokinase and tenecteplase. Another issue with reteplase that should be mentioned is its dose regimen. There is no recommended dosing for stroke treatment so far, and the dose regimen applied to our model may not be the best choice. It has been suggested that reteplase can achieve fast lysis due to its low fibrin affinity, which enhances its diffusion to the middle of the clot [43]. Our simulation results did not reveal the same. The transport of drugs in the clot is shown in Figure 4. The concentration distribution of reteplase along the length of the clot after the first bolus is similar to that of other drugs. Moreover, the lysis of binding sites for the four drugs started at a similar time at each point of the clot (Figure 7). Reteplase did not activate the lysis earlier than the other drugs in the middle of the clot. A possible reason is that the free-phase reteplase is depleted too fast to diffuse through the clot. Furthermore, the relation between the drug temporal concentration and lysis rate can be seen clearly from Figure 4 and Figure 7c. The lysis rate is much slower after 40 min, while reteplase concentration in the clot drops to a low level of 0.01 M. Our simulation results suggest that a higher initial dose or intra-arterial administration may improve the efficacy of reteplase. On the other hand, low drug concentration and slow kinetics help to keep the FBG concentration at a constant level and ensure a relatively low risk of ICH. Reteplase also has a good ability to resist PAI-1 inhibition (Figure 8 and Figure 9c) due to the removal of the protease domain of the molecules.

## 5. Conclusions and Further Perspectives

IV thrombolysis of four drugs has been simulated using the 1D mathematical model developed in our previous study. Our model is able to predict the therapeutic performance of alteplase, tenecteplase, reteplase, and urokinase based on their properties and mechanisms of action. The simulation results show that urokinase has the quickest recanalization rate but the highest ICH risk, in accordance with clinical findings in the literature. Moreover, our simulation of the tenecteplase therapy reveals its advantage over alteplase in providing a lower ICH risk and higher resistance to PAI-1, suggesting that it should be considered as a promising candidate for IV thrombolysis in AIS. Reteplase, on the other hand, is too slow to provide comparable lysis efficacy to other drugs, although it has the lowest ICH risk among the four drugs. The promising results obtained with our computational model encourages further studies of more complicated therapies, such as dual thrombolytic therapy with pro-urokinase and alteplase [45,46,47]. Given the unpredictable risk of clinical studies for AIS, our computational model can provide a useful tool to optimize the dose regimen for combinational therapies of multiple drugs and newly developed drugs if the PKPD mechanisms and reaction kinetics are known.

## Figures and Tables

**Table 1:** Parameters for the simulated drugs in plasma-phase reactions.

	Alteplase [19]	Urokinase (uPA)	Tenecteplase (TNK-tPA)	Reteplase
Initial concentration *C*_0_	0.05 nM	0.7 nM [25]	0 nM	0 nM
*K_M_*	28 μM	50 μM [26]	20 μM [27]	0.2 μM [28]
*k_1,cat_*	0.3 s^−1^	1 s^−1^ [26]	0.04 s^−1^ [27]	3.3 × 10^−4^ s^−1^ [28]
*k_5_*	37 μM^−1^s^−1^	160 μM^−1^ [29]	0.15 μM^−1^s^−1^ [30]	37 μM^−1^ s^−1^ [28]
*k_el_*	2.27 × 10^−3^s^−1^	4.06 × 10^−4^s^−1^ [31]	3.89 × 10^−4^s^−1^ [32]	8.33 × 10^−4^s^−1^ [33]
*k_cp_*	3.1 × 10^−4^s^−1^	4.39 × 10^−4^s^−1^ [31]	1.1 × 10^−4^s^−1^ [32]	18.2 × 10^−5^s^−1^ [33]
*k_pc_*	3.34 × 10^−4^s^−1^	1.28 × 10^−4^s^−1^ [31]	1.37 × 10^−4^s^−1^ [32]	1.53 × 10^−4^s^−1^ [33]
*V_c_* per body weight	0.057 L/kg	0.13 L/kg [31]	0.0496 L/kg [34]	0.25 L/kg [33]

**Table 2:** Kinetic parameters for the simulated drugs in clot-phase reactions.

	Alteplase	Tenecteplase	Reteplase	Urokinase
*k_a_*	0.01 μM^−1^s^−1^ [18]	0.01 μM^−1^s^−1^	0.01 μM^−1^s^−1^	0
Dissociation constant *k_d_*	0.58 μM	0.15 μM [34]	1.1 μM [35]	0
*k_d_,tPA*	0.0058 s^−1^	0.0015 s^−1^ [34]	0.011 s^−1^ [35]	0
*K_M_*	0.16	2.8 [27]	4.6 [28]	0.81 μM [36]
*k_M,cat_*	0.3	0.54 [27]	0.32 [28]	2.6 s^−1^ [36]

**Figure A1:**
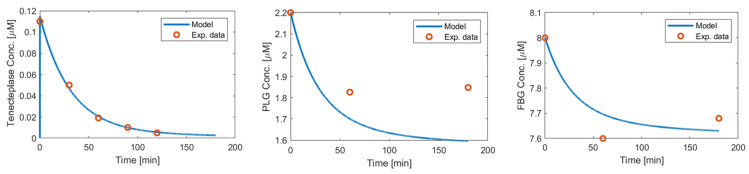
Comparison between simulation results and clinical results from TIBI 10B trial [38].

**Figure A2:**
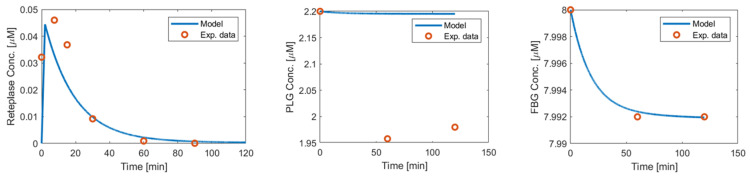
Comparison between simulation results and clinical results from Ulrich et al. [37].

**Figure A3:**
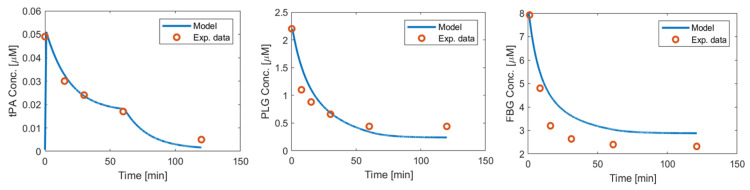
Comparison between simulation results and clinical results from Joerg et al. [31].

## Data Availability

Data available within the article or Appendix A.

## Appendix A. Comparison with Clinical Data in the Literature

For each drug, we compared the systemic PK simulation results with the clinical data of plasminogen activator, PLG, and FBG plasma concentrations from the literature. Model validation for alteplase can be found in our previous study [22].

### Appendix A.1. Tenecteplase

Comparisons were made with the clinical data reported in the TIBI 10B clinical trial [38] with a 30 mg single-bolus TNK-tPA thrombolysis therapy in 886 acute myocardial infarction patients. The predicted fibrinogen and tenecteplase plasma concentrations are in good agreement with the clinical results. The predicted plasminogen concentration is 10% lower than the clinical result at 180 min.

### Appendix A.2. Reteplase

Comparisons were made with the data reported in a dosing-range study in 18 healthy volunteers with a single 5.5MU intravenous bolus injection over 2 min [37]. The specific activity of reteplase (BM 06.022) in this case is 0.578 mg/MU. Our simulation results show good agreement with the clinical results in terms of reteplase and FBG plasma concentrations. The predicted PLG concentration is 20% higher than the clinical data at 120 min. However, the good match of FBG and reteplase plasma concentrations to the clinical data indicates that there is no need to further modify the kinetic parameters. The standard deviations of the clinal results should also be considered when making such comparisons.

### Appendix A.3. Urokinase

Comparisons were made with data reported in a pharmacokinetic study of urokinase therapy for acute myocardial infarction [31]. Twelve patients with myocardial infarction were dosed with an 11.3 mg urokinase bolus +11.3 mg IV infusion in 60 min. The predicted fibrinogen concentration is slightly higher than the clinical result. In order to keep the consistency of the reaction kinetic parameters between fibrinogen and plasmin applied in all four models, the parameters were not adjusted.
